# The essential schistosome tegumental ectoenzyme SmNPP5 can block NAD-induced T cell apoptosis

**DOI:** 10.1080/21505594.2020.1770481

**Published:** 2020-05-22

**Authors:** Catherine S. Nation, Akram A. Da’Dara, Patrick J. Skelly

**Affiliations:** Molecular Helminthology Laboratory, Department of Infectious Disease and Global Health, Cummings School of Veterinary Medicine, Tufts University, North Grafton, MA, USA

**Keywords:** Schistosoma, NAD, purinergic signaling, Tregs, immunomodulation

## Abstract

Infection with intravascular platyhelminths of the genus *Schistosoma* can result in the debilitating disease schistosomiasis. Schistosomes (blood flukes) can survive in the host for many years. We hypothesize that proteins on their host-interactive surface modify the worm’s external environment to help insure worm survival. Previously, we have shown that a surface ectoenzyme of *Schistosoma mansoni*, SmNPP5 – a nucleotide pyrophosphatase/phosphodiesterase – can cleave ADP and block platelet aggregation *in vitro*. In this work, we show that both adult schistosomes and recombinant SmNPP5 can cleave the exogenous purinergic signaling molecule nicotinamide adenine dinucleotide (NAD). In doing so, worms and rSmNPP5 can prevent NAD-induced apoptosis of T cells *in vitro*. Since regulatory T cells (Tregs) are especially prone to such NAD-induced cell death (NICD), we hypothesize that schistosome cleavage of NAD promotes Treg survival which creates a more immunologically hospitable environment for the worms *in vivo*. In addition to SmNPP5, schistosomes express another host-interactive NAD-degrading enzyme, SmNACE. We successfully suppressed the expression of SmNPP5 and SmNACE (singly or together) using RNAi. Only SmNPP5-suppressed worms, and not SmNACE-suppressed worms, were significantly impaired in their ability to cleave exogenous NAD compared to controls. Therefore, we contend that ectoenzyme SmNPP5 on the surface of the worm is primarily responsible for extracellular NAD cleavage and that this helps modulate the host immune environment by preventing Treg cell death.

## Introduction

Schistosomiasis is a highly debilitating disease afflicting over 200 million people worldwide while over 800 million live at risk of infection [1,2]. The disease is caused by intravascular platyhelminths, or blood flukes. Three major species infect humans: *Schistosoma mansoni, S. japonicum* and *S. haematobium* [3]. The larval form of this waterborne parasite, the cercaria, emerges from an infected freshwater snail (intermediate) host and penetrates the skin of a mammalian (definitive) host. Here it differentiates into a schistosomulum which enters a blood vessel, migrates through the heart and lungs, and ultimately reaches the hepatic portal vein. Here the parasites mature into adults. These sexually dimorphic worms then pair and migrate through the bloodstream to the mesenteric veins (*S. mansoni* and *S. japonicum*) or the vesical venous plexus of the bladder (*S. haematobium*) [4]. Adult females can lay hundreds of eggs each day. The host’s immune response to these eggs is responsible for the pathology of infection which can impact the liver, gastrointestinal tract, spleen, kidneys, urinary tract and uterus; in chronic and heavy infections morbidity can be severe [1,5]. Adult worms can survive for many years in their hosts where they modulate the vascular environment and the immune response [6–10].

We hypothesize that host interactive proteins expressed at the schistosome surface are central to the worm’s ability to modulate the local environment and ensure survival [6]. Among these surface exposed proteins of *Schistosoma mansoni* is the tegumental enzyme SmNPP5 (GenBank accession number, ACI29972). This is a 53 kDa protein that belongs to the ecto-nucleotide phosphodiesterase/pyrophosphatase (E-NPP) family of enzymes. The SmNPP5 gene is rapidly turned on in the intravascular parasitic life stages, following invasion of the definitive host [11]. Larval parasites (schistosomula) whose SmNPP5 gene is suppressed are significantly impaired in their ability to establish infection in experimental animals and the protein is considered a virulence factor for the worms [11]. We have found that recombinant SmNPP5 can cleave adenosine diphosphate (ADP) and can inhibit platelet aggregation in a dose dependent manner, as measured by multiple electrode aggregometry (MEA) using whole blood [12].

A second important signaling molecule in the extracellular environment is nicotinamide adenine dinucleotide (NAD). NAD is a vital molecule found in all living cells where it engages in hundreds of biochemical reactions through its role in redox and other reactions. In recent years NAD has also become recognized as an extracellular signaling metabolite that is important in cell-cell communication [13,14]. Under normal conditions, NAD is present in very low levels in serum; however in response to mechanical or oxidative stress it is released in higher levels into the extracellular space [15,16]. Here, NAD serves as a damage associated molecular pattern (DAMP), that can modulate host immunity [15,17]. For instance, during inflammation, extracellular NAD acts to modulate T cell homeostasis by driving the process of NAD-induced T cell death (NICD) [18]. This process occurs through the action of T cell surface mono-ADP ribosyltransferase (ART2) that catalyzes the transfer of the ADP-ribose (ADPR) group from NAD to the cell surface ATP receptor P2X7 (P2X7R) [19]. This leads to P2X7R activation which induces apoptosis, a process characterized by shedding of the cell adhesion molecule L-selectin (CD62L), exposure of phosphatidylserine (PS), formation of membrane pores, and ultimately cell death [18]. Relatively low levels of NAD (1–10 µM) suffice to induce NICD [15,20].

CD4^+^ T cells are commonly divided into regulatory T cells (Tregs) and conventional T helper (Th) cells. Tregs, defined as CD4^+^CD25^+^FoxP3^+^ T cells that act to suppress potentially deleterious activities of Th cells, are particularly sensitive to NICD because of their relatively high expression of the P2X7 receptor [21]. This is intriguing in the context of schistosomiasis since it is known that schistosome infection leads to the expansion of Tregs that play key roles in immunoregulation [22,23]. Because of this, we are interested in any ability schistosomes may have to control levels of extracellular NAD since this could have a bearing on Treg levels during worm infection. Since human homologs (hNPP5 and hNPP1) of the parasite ectoenzyme SmNPP5 mentioned above can cleave NAD [24,25], we set out to determine if SmNPP5 likewise has this capability. We report here that recombinant SmNPP5 can indeed hydrolyze NAD.

Earlier it was reported that a different schistosome tegumental ectoenzyme could cleave exogenous NAD [26]. This enzyme – *S. mansoni* NAD cleaving enzyme (or SmNACE, GenBank accession no. AAX35328) – is a 35kDa GPI-linked glycoprotein that is a member of the ADP-ribosyl cyclase family [26]. However, unlike other ADP-ribosyl cyclases, SmNACE does not demonstrate appreciable cyclase activity, generating just trace amounts of cADPR following NAD cleavage [26,27]. The SmNACE gene, like the SmNPP5 gene, has highest relative expression in the adult worm life stages and both proteins are expressed in the outer tegument and exposed on the surface of the adult worms [11,26] where they could potentially modulate extracellular NAD levels.

In this work we examine the ability of adult schistosomes and SmNPP5 to cleave NAD and impact NICD. We further compare the roles of SmNPP5 and SmNACE on NAD cleavage using RNA interference to knock down the expression of both genes in adult schistosomes. Our aim is to clarify ways in which schistosomes may modulate the host immune environment to promote their long-term survival.

## Materials and methods

### Parasites and mice

*Biomphalaria glabrata* snails (strain NMRI) infected with *Schistosoma mansoni* were obtained from the Schistosomiasis Resource Center (Biomedical Research Institute (BRI)). Cercariae were obtained from infected snails [28] and used to infect Swiss Webster mice (at ~100 cercariae/mouse). Seven weeks post-infection, adult male and female worms were recovered from the infected mice by vascular perfusion and cultured in complete Dulbecco’s modified Eagle’s medium (DMEM)/F12 medium supplemented with 10% heat-inactivated fetal bovine serum, 200 U/mL penicillin and 200 μg/mL streptomycin, 0.2 μM Triiodo-L-thyronine, 1 μM serotonin, 200 μM ascorbic acid and 10 μg/mL human insulin, and were maintained at 37°C, in an atmosphere of 5% CO_2_ [29]. Lymph nodes and spleens were removed from 6-8-week-old female Balb/C mice for T cell purification (as described below). All protocols involving animals were approved by the Institutional Animal Care and Use Committees (IACUC) of Tufts University.

### NAD cleavage by adult worms

Cleavage of NAD is conveniently measured using 1,N^6^-etheno-NAD (ε-NAD, Sigma, N2630) since hydrolysis of this molecule can be detected fluorometrically (with excitation at 300 nm and emission at 410 nm) [26,30]. Live adult worms (individually or in groups of two) were washed 3x with assay buffer (50 mM Tris-HCl [pH 9.0], 120 mM NaCl, 5 mM KCl, 50 mM glucose and 2 mM CaCl_2_) then incubated in assay buffer containing 500 µM ε-NAD in a final volume of 200 µl at 37°C. Changes in fluorescence were followed over time using a Synergy HT microplate reader (BioTek). The minimal background (substrate-only control) values have been subtracted from all data points which are reported as relative fluorescence units (RFU) per individual worm.

### NAD cleavage by recombinant SmNPP5

Recombinant SmNPP5, expressed in suspension adapted FreeStyle Chinese Hamster Ovary Cells (CHO-S), was purified from culture medium by standard immobilized metal affinity chromatography (IMAC) using HisTrap Excel columns, as described previously [12]. The ability of rSmNPP5 to cleave NAD was measured by two methods. In the first method, recombinant enzyme (12.5–50ng) was incubated with ε-NAD essentially as described above but at room temperature (RT). The second method employed β-NAD (Roche, 10127965001); after incubating 1 μg SmNPP5 with β-NAD (2 mM) in 500 μl enzyme assay buffer for either 1- or 5-hours at 37°C, calf intestinal alkaline phosphatase (CIP, at 70 u/mL, Promega, M182A) was subsequently added to samples of each reaction mixture for 15 min at RT. Any inorganic phosphate (Pi) released was then measured using a Phosphate Colorimetric Assay Kit (BioVision, K410), following the manufacturer’s instructions. Absorbance (650 nm) was measured using a Synergy HT microplate reader (Biotek).

### Thin layer chromatography (TLC) analysis

The products from some enzymatic reactions were analyzed using TLC Silica gel 60 F_245_ (aluminum sheet, 20 × 20 cm, EMD Milipore, 105554). Aliquots from enzyme reaction mixtures, as well as chemical standards β-NAD (Roche), AMP and NMN(Sigma, A2252 and N3501),were spotted onto a TLC sheet and dried. Separation was achieved using a mobile phase composed of n-butanol:acetone:acetic acid (glacial):ammonia (5%):water (45:15:10:10:20) and analytes were visualized under UV at 254 nm [31].

### T cell purification

Pooled lymph nodes (inguinal, popliteal, cervical, axillary, and mesenteric) and spleens were dissected from Balb/c mice and placed in ice cold Hank’s Balanced Salt Solution (HBSS) containing 2% heat inactivated Fetal Bovine Serum (FBS). Cell suspensions were prepared by passing the tissue through a 40 µm cell strainer. Lymphocytes were collected by centrifugation at 300 x g for 5 minutes at 4°C and resuspended in RPMI. Splenocytes were collected by centrifugation in the same manner, resuspended in Red Blood Cell Lysing Buffer (Sigma, R7757) for two minutes at room temperature, washed by centrifugation and, finally, resuspended in RPMI. Cell numbers and viability were determined by diluting 1:10 with 2% Trypan Blue for 5 minutes followed by counting on a hemocytometer.

T cells were isolated from the cell mixture using a Dynabeads Untouched Mouse T cell kit (Invitrogen, 11413D) following the manufacturer’s instructions. T cell purity was verified by cell staining using phycoerythrin-conjugated anti-CD45 R/B220 antibody (BD Biosciences, 553090) and FITC-conjugated anti-CD3 antibody (BD Biosciences, 553061), followed by flow cytometry, as previously described [19,20]. T-cell purity was consistently >95%.

### Measuring the impact of schistosomes and rSmNPP5 in an NAD-induced cell death (NICD) assay

To measure the effect of NAD on T cell survival, β-NAD (500 µM) was incubated at 37°C alone or together with adult schistosome parasites (6 male and female pairs in 500 µl assay buffer). Samples were collected after 2 and 24 hours and were added to cultures of isolated T cells (2.5x10^5^cells/tube) at a concentration of 25 µM NAD (based on initial concentration). To measure the percentage of cells that were apoptotic after 30 min, all cells were washed once with Annexin Binding buffer (0.1 M Hepes pH 7.4, 1.4 M NaCl, 25 mM CaCl2), then stained with FITC-conjugated Annexin V (1ug/ml, BD Biosciences, 560931) and propidium iodide (10ug/ml, BD Biosciences, 556463) in Annexin Binding buffer for 20 minutes at room temperature and protected from light. Next, cells were washed twice with flow cytometry buffer (PBS, 1% BSA, 0.05% NaN_3_) and resuspended in 100 µl buffer before being subjected to flow cytometry using a BD Acurri C6 cytometer (BD Biosciences). Additional T cell incubations included in this experiment were: no addition of NAD (as a control) as well as incubation with medium in which worms (without NAD) were kept for 2 or 24 h (to test for the impact of any worm products on T cell viability). Data obtained using T cells isolated from lymph nodes and spleensare combined.

A similar scheme to that just described was used to monitor the impact of recombinant SmNPP5 on NICD. Briefly, 500 µl of 1 mM β-NAD in assay buffer was incubated with 1 µg purified rSmNPP5 at 37°C. Aliquots were recovered one hour and five hours later and added to purified T cells (at 25 µM, based on starting concentration) which were then processed as described above.

### RNA interference

Adult worms were treated with a synthetic siRNA (average of ~ 6 µM) targeting either SmNPP-5 (SmNPP-5 siRNA1: 5ʹ-TTGATGGATTTCGTTATGATTACTTTG-3ʹ) or SmNACE (1:1 mix of SmNACE siRNA1: 5ʹ-CCAGGATATCTGTTTGATGAATTGA-3ʹ and SmNACE siRNA2: 5ʹ-AGGTTGAACATGGAGCTACTAATAT-3ʹ) or with both SmNPP5 and SmNACE siRNAsor with control siRNA (5ʹ-CTTCCTCTCTTTCTCTCCCTTGTGA-3ʹ) predicted to target no gene in the *S. mansoni* genome. Delivery of siRNAs to the parasites was by electroporation in Gene Pulser Electroporation Buffer (BioRad, 1652676) as described previously [32]. Gene suppression was assessed 7 days post-treatment by comparing mRNA levels (using RT-qPCR) and enzyme activity (i.e. ε-NAD cleavage capability, using the protocol described above).

### Gene expression analysis

The levels of expression of the SmNPP5 and SmNACE genes were determined by reverse transcription quantitative PCR (RT-qPCR), using a custom TaqMan gene expression system (Applied Biosystems). RNA was isolated from siRNA-treated worms using Trizol reagent (Invitrogen, 15596026) according to the manufacturer’s instructions. Residual DNA was removed by DNase digestion using a TurboDNA-free kit (Invitrogen, AM1907). cDNA was synthesized using 850ng RNA and TaqMan Reverse Transcription Reagents (Applied Biosystems, N8080234). Final reactions contained 0.8 mM MgCl_2_, 1 mM dNTP mix, 20 U RNase inhibitor, 50 U MultiScribe reverse transcriptase, and 2.5 mM DTT (Invitrogen, P2325).

Primer sets and reporter probes labeled with 6-carboxyfluorescein (FAM), obtained from Applied Biosystems, were used for RT-qPCR to determine levels of target gene expression. To detect SmNACE expression, the following primers and probe were used: Forward Primer: 5ʹ-TCCTCGAGTACAACAATTAACAGTGAAATT-3ʹ; Reverse Primer: 5ʹ-TTCTTGCAGACTCCACGATTCA; Probe: 5ʹ-FAM-CATGTTTGTCGGTTATTTAC-3ʹ. SmNPP5 and alpha tubulin (endogenous control) primers and probes have already been reported [33]. Each RT-qPCR was performed using cDNA equivalent to 85 ng total parasite RNA according to the manufacturer’s universal conditions PCR protocol, in a final volume of 20 μl. All samples were run in triplicate and underwent 40 amplification cycles on a Step One Plus Real Time PCR System Instrument (Applied Biosystems). For relative quantification, the ΔΔCt method was employed [34].

### Statistical analysis

Statistical analysis was carried out using Graphpad Prism 8 for Mac OS X (Graphpad Software). One-way ANOVA with Tukey’s multiple comparison’s test was used for to assess apoptosis data (at each time point) and NAD cleavage by rSmNPP5 and worms. Two-way ANOVA was used to assess differences in ε-NAD cleavage among siRNA treated groups and between males and females. *p *< 0.05 values were considered significant.

## Results

### Adult schistosomes cleave NAD

**Figure 1.**
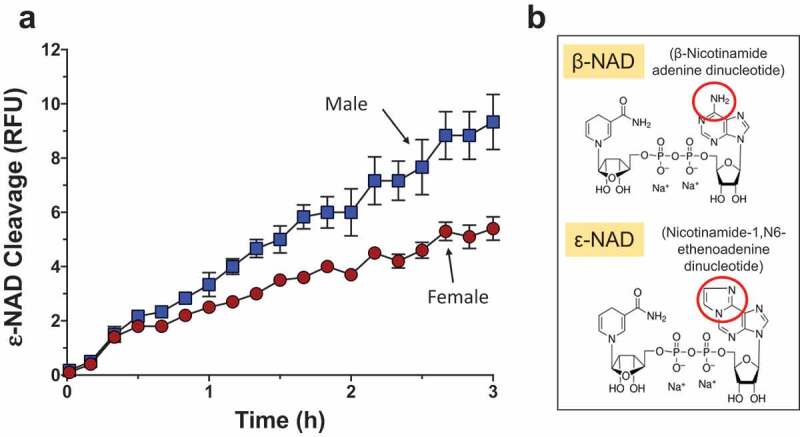
Live adult schistosomes cleave ε-NAD. a. Male (blue squares) or female (red circles) worms, as indicated, were incubated with ε-NAD and mean fluorescence (±SEM) was measured over time, indicating cleavage. *p *< 0.001, 2-Way ANOVA, n ≥ 3. b. Structures of β-NAD (top) and ε-NAD (bottom); red circles indicate differences between the chemicals.

To test the ability of live schistosomes to hydrolyze exogenous NAD, individual male and female parasites were incubated with ε-NAD (1,*N*^6^-etheno-NAD) *in vitro* (Figure 1a). ε-NAD is a membrane impermeable, synthetic substrate that fluoresces when hydrolyzed; cleavage breaks the quenching interaction between the ε-adenine and pyridine ring of nicotinamide [30,35]. The chemical structures of both β-NAD (the natural form of this compound) and ε-NAD are shown in Figure 1b with red circles highlighting their differences. Male parasites (blue boxes, Figure 1a) exhibit significantly higher activity than female parasites (red circles) (2-way ANOVA *p *< 0.001, Figure 1a).

### Adult schistosomes prevent NAD-induced cell death in vitro

**Figure 2.**
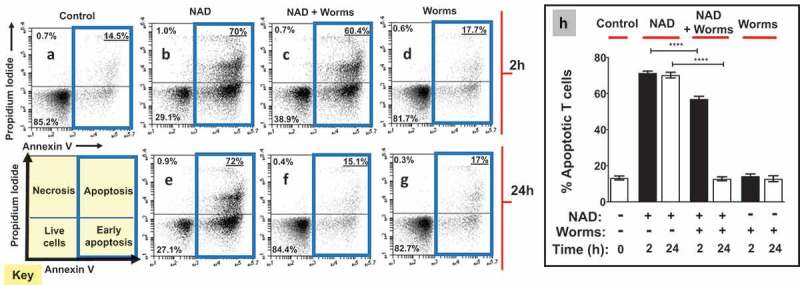
Adult worms prevent NAD induced T cell death (NICD) *in vitro*. Panels athrough gare representative (from one run) flow cytometry dot plots of purified T cells following various experimental treatments (described below) and panel h(right) represents composite data showing the mean percent total apoptotic T cells (± SEM) following these treatments in replicate. NAD was incubated at 37°C for 2 or 24 h with (panels cand f) or without (panels band e) adult schistosome parasites (male and female pairs). Equivalent amounts of NAD (25 µM, based on initial concentration) were added to cultures of purified T cells (2.5x10^5^cells/tube) for 30 min. Cells were then stained and subjected to flow cytometry. Controls include T cells incubated without NAD (Control, panel a) as well as cells incubated with worm culture medium that did not contain NAD (Worms, panels dand g). The “Key” at lower left indicates the cell status in each FACS plot quadrant (“live”, “early apoptotic”, “apoptotic” and “necrotic”). Panels b- dshow samples tested after the 2 h incubation period and panels e- gafter the 24 h incubation period. Of primary interest here, the percentages of total apoptotic cells (i.e. early plus late apoptosis; right upper and lower quadrants) are bounded by blue boxes in panels a- g. ****p < 0.0001 NAD v NAD + worms (one-way ANOVA at each time point), n ≥ 4 in each case.

To assess the impact of adult worms on NAD-induced cell death (NICD), flow cytometry was used to determine the status of purified T cells treated with NAD that had been previously incubated in the presence or absence of adult worms. The hypothesis being tested is that NAD alone will induce T cell apoptosis but, following its incubation with the NAD-degrading worms, this effect will be ablated. The status of the T cells was assessed by flow cytometry after staining with FITC conjugated Annexin V and propidium iodide. Four populations of cells were identified: live cells, early apoptotic cells, late apoptotic cells and necrotic cells. (A “cell status” key is depicted in the lower left panel of Figure 2). Representative flow cytometry images (from one run) are shown in Figure 2, panels A through G for all treatments. We are interested in the percent overall apoptotic and, for all treatments, this cell population is bounded by blue boxes in Figure 2, panels A – G. Data from experimental replicates are combined and mean percent apoptotic values (±SEM) are presented in Figure 2h.

Control cell populations untreated with NAD are largely healthy (Figure 2a) and have a low percent of apoptotic T cells (Mean 13 ± 1% overall apoptotic, “Control” Figure 2h). However, following treatment with NAD that had been incubated in medium for 2 h (representative Figure 2b), the mean percent apoptotic increases to 71 ± 1% (“NAD”, Figure 2h). Likewise, adding NAD that had been incubated in medium for 24 h (representative Figure 2e) induces apoptosis to about the same extent (mean value of 70 ± 2%, “NAD”, Figure 2h). In contrast, preincubation of NAD with adult schistosomes for 2 hours prior to being added to T cell cultures (“NAD + worms”), significantly reduced the mean percent of apoptotic T cells (57 ± 1% Figure 2h; representative Figure 2c). In addition, preincubation of NAD with schistosome parasites for 24 hours prior to being added to T cells completely abrogated the ability of NAD to induce T cell apoptosis (13 ± 1% apoptotic Figure 2h, representative Figure 2f) (*p*<0.001 at the 2- and 24-hour time points, ANOVA). Note that control medium that contained worms (and no NAD) for 2 h (representative Figure 2d) or 24 h (representative Figure 2g) does not, by itself, induce apoptosis; the mean percent total apoptotic here issimilar to that of control cells (14 ± 1% after 2 hours, 13 ± 2% after 24 hours, “worms”, Figure 2h).

### rSmNPP5 can degrade NAD

**Figure 3.**
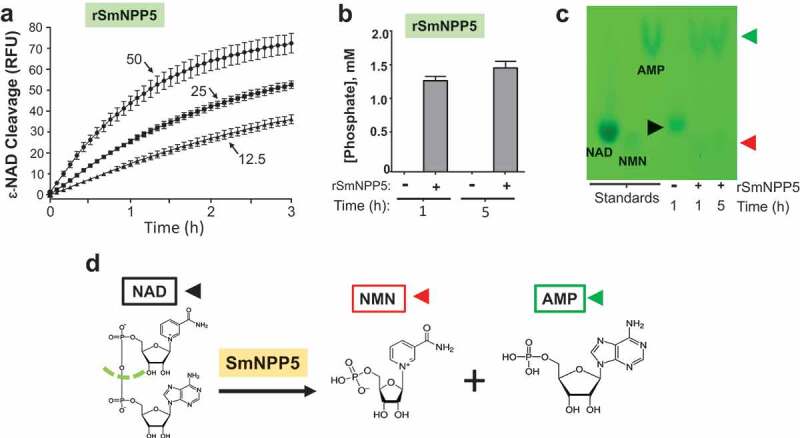
Purified recombinant SmNPP5 cleaves NAD. a. ε-NAD was incubated with 12.5, 25, or 50 ng of rSmNPP5, as indicated, and cleavage was assessed by an increase in fluorescence over time (n = 3, mean ± SEM) b. β-NAD was incubated with (+) or without (-) 1 µg rSmNPP5 for either 1 h or 5 h (as indicated) and phosphate levels (mean ±SEM) were assessed following CIP treatment. c. Thin Layer chromatography analysis of reaction products following NAD (black arrowhead) incubation with (+) or without (-) 1 µg rSmNPP5 for 1 h or 5 h (as indicated). Cleavage yields NMN (red arrowhead) and AMP (green arrowhead). Migration of standards is shown at left. NAD, nicotinamide adenine dinucleotide; NMN, nicotinamide mononucleotide; AMP, adenosine monophosphate. d. Depiction of NAD cleavage reaction catalyzed by SmNPP5; structures of NAD and cleavage products NMN and AMP are shown.

To determine if rSmNPP5 could cleave NAD, recombinant enzyme was incubated with ε-NAD and fluorescence was measured over time. Figure 3a shows that rSmNPP5 (tested at 12.5 and 25 and 50 ng, as indicated) can hydrolyze this substrate. To confirm these results, in a second test, rSmNPP5 was incubated with β-NAD at 37°C and aliquots were taken after 1 and 5 hours. As described in Methods, samples were then treated with calf intestinal phosphatase (CIP) and phosphate levels were subsequently assessed. Figure 3b Shows that phosphate is only detected following the incubation of NAD with rSmNPP5 (+rSmNPP5) at both time points and not in the absence of the recombinant enzyme (-rSmNPP5). This means that SmNPP5 has exposed terminal phosphates that become accessible to the action of CIP and confirms that SmNPP5 cleaves NAD. Under these conditions, 1358 ± 82 µM phosphate was released after 1 h (68% of total), increasing to 1546 ± 143 µM by 5 h (77% of total), while no phosphate was present in the control samples (-SmNPP5 enzyme) at the same time points.

Thin layer chromatography (TLC) was used to identify the products of SmNPP5-mediated cleavage of NAD. Figure 3c shows a TLC silica gel sheet revealing the pattern of migration of NAD (black arrowhead) before (-) and after (+) 1 h or 5 h incubation with rSmNPP5 (as indicated). Migration patterns of relevant standards (NAD, nicotinamide mononucleotide (NMN) and adenosine monophosphate (AMP)) are shown on the left side of the sheet. It is clear that SmNPP5 cleavage of NAD yields NMN (red arrowhead) and AMP (green arrowhead). Figure 3d depicts the chemical reaction catalyzed by SmNPP5, as revealed by this experiment; the cleavage site on NAD is depicted by a green dashed line and the reaction products (NMN and AMP) are shown.

### SmNPP5 prevents NAD-induced cell death (NICD) in vitro

**Figure 4.**
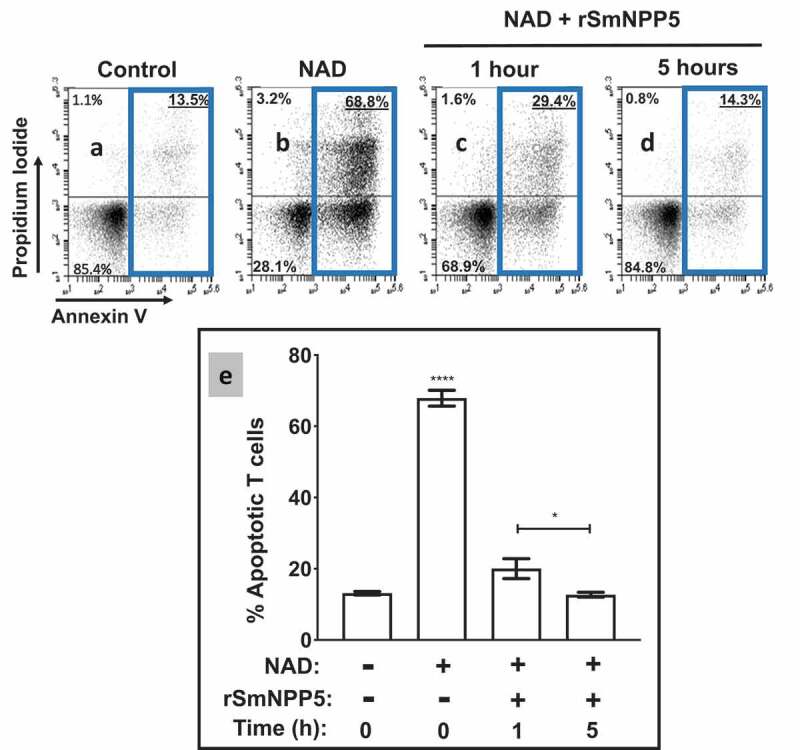
Recombinant SmNPP5 prevents NAD induced T cell death (NICD)*in vitro*. Panels athough dare representative flow cytometry dot plots of purified T cells following exposure to NAD (panel b) or not (Control, panel a) or to NAD that had been incubated with1 µg rSmNPP5 for 1 hour (panel c) or for 5 hours (panel d). The “Key” depicted in Figure 2 (lower left) applies here too; the percentages of total apoptotic cells are bounded by blue boxes. Panel erepresents composite data from replicate experiments, showing the mean percent total apoptotic T cells (± SEM) following the treatments indicated. *p<0.05 1 h v 5 h, ****p<0.0001+ NAD at time 0 v all other groups, One-way ANOVA, n≥4 in each case.

Having confirmed that rSmNPP5 can degrade NAD, the impact of this cleavage on NICD was assessed using essentially the same protocol as described above for whole worms. T cells were purified (>95% purity as determined by flow cytometry) and then incubated with NAD (for 30 min) that either had, or had not, been incubated with rSmNPP5 at 37°C for 1 or 5 h. Cells were then stained with FITC conjugated Annexin V and propidium iodide (PI) and subjected to flow cytometry to assess the percent of apoptotic T cells. Representative plots are shown in Figure 4a-d; data from experimental replicates are combined and mean percent overall apoptotic values (±SEM) are presented in Figure 4e. As before, the percent of apoptotic control cells (not exposed to NAD) is low (13 ± 0.5% of total cells). As expected, following treatment with NAD, a significant percentage of T cells become apoptotic (67.9 ± 2%, Figure 4e *p*<0.0001). However, following incubation with rSmNPP5 for 1 hour (Figure 4, “+ rSmNPP5”), this population is significantly reduced (to 20 ± 3% of cells, *p*<0.001 compared to “-rSmNPP5”, Figure 4e). After 5 h incubation with rSmNPP5, only 12.7 ± 0.6% of cells are apoptotic- similar to untreated control cells, and significantly lower than the 1-hour treatment group (*p*<0.05). It is clear that, as seen following worm preincubation with NAD, rSmNPP5 preincubation with NAD likewise prevents NICD *in vitro*.

### SmNPP5 is responsible for NAD cleavage by live adult worms

**Figure 5.**
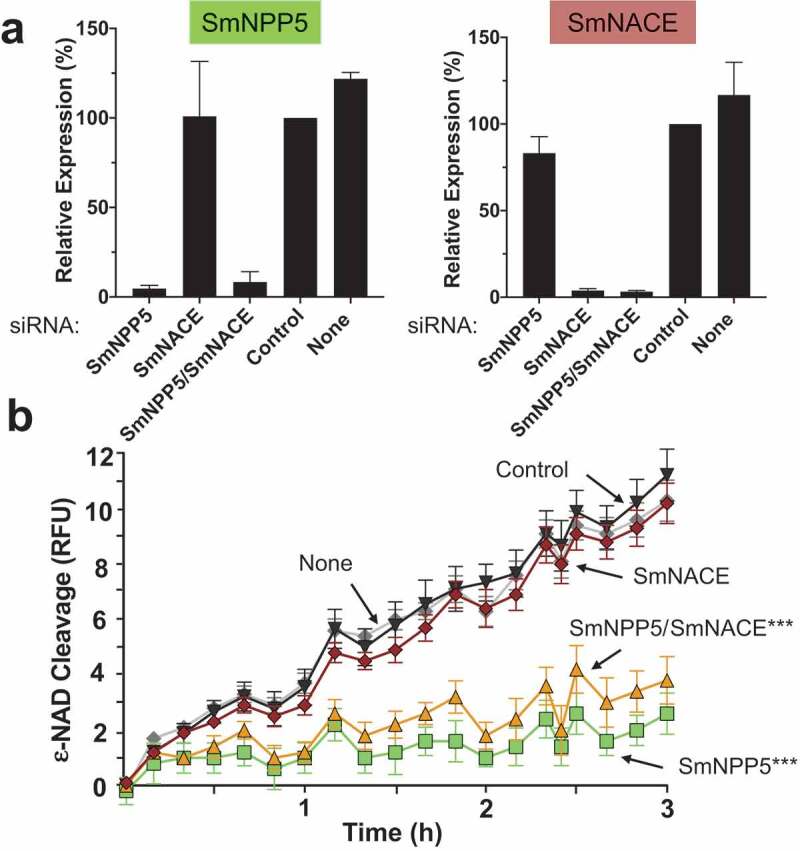
Knockdown of SmNPP5, but not SmNACE, reduces the ability of adult schistosomes to cleave extracellular ε-NAD. Adult male worms were treated with the indicated siRNAs. Seven days later the mRNA levels of SmNPP5 (left) and SmNACE (right) were assessed by RT-qPCR, shown in a. Average expression level (±SEM) is reported relative to the Control treatment, set at 100%. In b, the ability of siRNA treated individual worms from each group to cleave ε-NAD (mean RFU ±SEM,) over time is shown. ***p<0.0001 for SmNPP5 or SmNPP5/SmNACE compared to Control, None, and SmNACE groups, 2-way ANOVA with Tukey’s multiple comparisons test, n≥5 for each group.

We show here that SmNPP5 can cleave NAD. A previous report identified another schistosome ectoenzyme – designated SmNACE – that can cleave NAD [26]. To assess the relative contributions of SmNPP5 and SmNACE to the ability of live worms to cleave NAD, we first suppressed the expression of the genes encoding these proteins using RNA interference. Adult male schistosomes were treated with siRNAs targeting SmNPP5 or SmNACE or both genes. Seven days later the levels of expression of these genes in these worms were compared with their expression levels in worms treated with a control siRNA or with no siRNA. Figure 5a shows that robust suppression of both genes was achieved, as assessed by RT-qPCR. Relative to the control group treated with an irrelevant siRNA (“Control”, Figure 5a), the expression of both target genes was suppressed by >95% in both the single and double knockdown groups.

To investigate whether this RNAi-mediated silencing of the SmNPP5 and SmNACE genes had any impact on the NAD cleavage activity of live schistosomes, the ability of adult male worms from each treatment group to cleave exogenous ε-NAD was assessed 7 days after siRNA treatment and results are shown in Figure 5b. Both of the control groups (i.e. those treated with the irrelevant siRNA (“Control”, Figure 5b) or with no siRNA, (“None”, Figure 5b) cleaved ε-NAD to a similar extent. SmNPP5 gene suppression resulted in a significantly impaired ability to cleave ε-NAD in both the single SmNPP5 knockdown and dual SmNPP5/SmNACE knockdown groups (*p*<0.0001 versus Control, None and SmNACE siRNA treatments). In contrast, SmNACE gene suppression did not appreciably impair the worm’s ability to cleave ε-NAD. The SmNACE-suppressed worms exhibited no significant reduction in their ability to cleave NAD compared with controls.

## Discussion

We are interested in understanding if and how schistosomes can control the biochemistry of their local environment. In this work we focus on molecular interactions between the worms and the host purinergic signaling molecule NAD. Here we confirm that live schistosomes can cleave this molecule. The physically larger males are observed to cleave more exogenous NAD compared to their smaller female counterparts. Such an ability may be selectively advantageous for the worms since extracellular NAD can exert immunomodulatory functions. Specifically, NAD has been shown to induce T-cell apoptosis. This phenomenon – NAD-induced cell death (NICD) – occurs when a cell-surface ADP ribosyl transferase (ART2) enzyme cleaves NAD and transfers a reaction product (ADPR) to the purinergic receptor P2X7 (P2X7R) inducing apoptosis [15]. The expression levels of ART2 and P2X7R at a cell’s surface dictate its sensitivity to NICD. Naïve T cells constitutively express both ART2 and P2X7R making them sensitive to NICD. In contrast, when T cells are activated, ART2 is proteolytically cleaved, rendering these cells resistant to NICD [36,37]. In contrast, regulatory T cells (Tregs) express high levels of P2X7R, rendering them exquisitely sensitive to NICD [15,16,21]. Consistent with this is the finding that injecting NAD into mice results in dramatic reductions of Tregs within 24 hours [18,21].

The impact of NAD on Treg survival is noteworthy in the context of schistosomiasis since it is known that schistosome infection leads to the expansion of Tregs that play key roles in immunoregulation [22,23]. Treatment of *S. japonicum* infected mice with a monoclonal antibody targeting Tregs (an anti-CD25^+^ mAb) led to a reduction in the Treg population which coincided with a reduced worm burden compared to untreated, infected control mice [38]. These data suggest a protective roll for Tregs in schistosome survival. Furthermore, the efficacy of an anti-*S. japonicum* vaccine has been shown to be substantially improved in mice whose Treg cell numbers have been decreased [39]. It has also been demonstrated that Tregs are involved in suppressing the granulomatous pathology induced by eggs during chronic infection [40], favoring host survival. Here, we confirm that NAD can indeed induce T cell killing but, following NAD preincubation with adult schistosomes for either 2 h or 24 h before being added to T cells in culture, this ability is blocked. We argue that by preferentially inhibiting Treg apoptosis (via NAD cleavage) schistosomes work to maintain a more immunotolerant environment for themselves *in vivo* [41,42].

We have characterized a collection of schistosome tegumental ectoenzymes with the ability to interfere with host purinergic signaling including SmNPP5 [6,12,33]. SmNPP5 is expressed exclusively in intra-mammalian parasite life stages [11]and highest gene expression is found in mated adult males where the protein is found predominantly in the dorsal tegument [43].

Given that human homolog hNPP5 (as well as human hNPP1 (PC-1)) can cleave NAD [24,25], we examined whether SmNPP5 likewise has this property. We found that recombinant SmNPP5, expressed in secreted form from transfected CHO cells and purified by IMAC, was able to degrade both β-NAD (the natural form of this molecule) as well as its fluorescent analog ε-NAD. Analysis using thin layer chromatography (TLC) showed that SmNPP5 cleaves NAD to yield NMN and AMP. Not surprisingly, preincubating NAD with rSmNPP5 before adding it to isolated T cells in culture was found to impede NICD and the longer the preincubation, the greater the impedance. Based on these findings, we hypothesized that schistosomes use tegumental SmNPP5 to degrade exogenous NAD and thus impede Treg apoptosis. However, previous work has shown that another schistosome ectoenzyme can cleave exogenous NAD [26], meaning that SmNPP5 is not the only tegumental enzyme with this ability. This second enzyme, designated *S. mansoni* NAD Cleaving Enzyme, or SmNACE, is a 35kDa GPI-linked glycoprotein that is a member of the ADP-ribosyl cyclase family; it exhibits 37% and 38% sequence similarity to human CD38 and CD157, respectively [26]. Like the SmNPP5 gene, the SmNACE gene too has highest relative expression in the adult worm life stages [26]. SmNACE cleaves NAD in a different manner compared to SmNPP5, largely yielding ADPR and nicotinamide [26].

To explore the relative importance of SmNPP5 and SmNACE for the worm’s ability to cleave NAD, we first used RNAi to suppress the expression of the genes encoding the enzymes (alone or together, by treatment with gene-specific siRNAs) and subsequently monitored the ability of the worms to hydrolyze exogenous NAD. While robust suppression of both genes was seen, only the SmNPP5-suppressed worms, but not the SmNACE-suppressed parasites, were significantly impaired in their ability to cleave added NAD. Thus, SmNPP5 is the principal NAD-cleaving ectoenzyme expressed by schistosomes. This result explains published reports that a selected chemical inhibitor of recombinant SmNACE-driven NAD-hydrolysis does not block the ability of live worms to hydrolyze NAD [44]; SmNACE inhibitors are not necessarily expected to also inhibit SmNPP5-driven NAD cleavage on worms.

This work shows that the *S. mansoni* virulence factor SmNPP5, previously shown to be an ADPase [12], is also an NADase. NADases are quite widespread as virulence factors among intracellular pathogenic bacteria [45]; many act as ADP-ribosyltransferases that cleave NAD and attach the hydrolysis product ADPR onto a target protein [46,47]. Others are NAD glycohydrolases that cause NAD level imbalance within the cell and enhance multiplication of intracellular pathogenic bacteria [48] and/or kill host cells [49,50]. The NADase of Group A *Streptococcus* has been shown to also exert an impact in the extracellular compartment where, by a mechanism that remains unclear, it can inhibit IL-1β release from human monocytes [51,52].

NAD and NAD-cleaving enzymes are also known to impact immunological function in other kingdoms of life. For instance, NAD triggers metabolic profiles in some plants that are associated with resistance to pathogens [53] and, in tomatoes, NAD has been reported to induce innate immune responses that protect the plants from infection with the root-knot nematode, *Meloidogyne hapla* [54]. Additionally, some immune receptors of plants are NADases that have been reported to promote resistance to pathogens [55].

**Figure 6.**
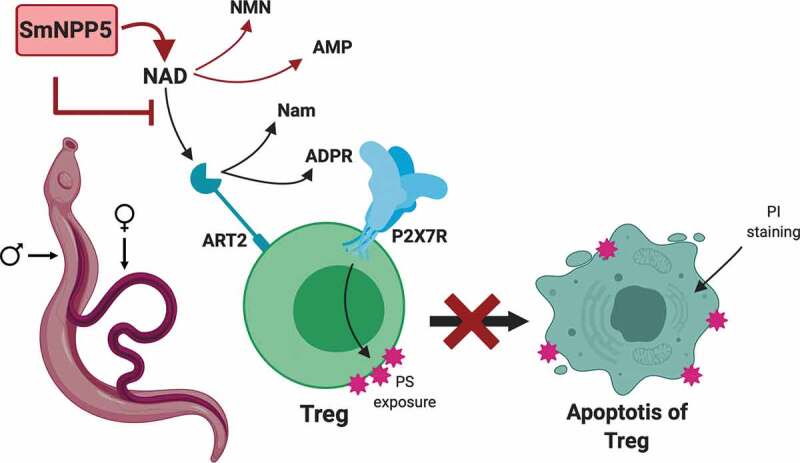
Diagrammatic representation of NAD induced T cell death (NICD) and its inhibition by rSmNPP5 and live schistosomes. A Treg cell is depicted (green) with its surface P2X7 receptor (P2X7R, blue) and ADPR ribosyl transferase ART2 (teal). NAD is a substrate for ART2 which generates nicotinamide (Nam) and ADPR; transfer of ADPR to P2X7R triggers apoptosis and this process results in exposure of phosphatidylserine (PS, pink star shapes) at the T cell surface. Cell membrane permeability increases which permits propidium iodide (PI) entry and signals late-stage apoptosis. Adult male and female schistosomes (left), through the action of surface ectoenzyme SmNPP5 (pink box), cleave extracellular NAD to NMN and AMP thus removing the ART2 substrate and preventing NAD induced Treg apoptosis.

In schistosomes, the NADase SmNPP5 acts in the extracellular environment and Figure 6 demonstrates one way that this ectoenzyme could act to increase the virulence of the intravascular worms. NAD is shown to be a substrate of the Treg receptor ART2 which cleaves it to generate nicotinamide (Nam) and ADPR. The latter is used to ribosylate the P2X7 receptor (blue) on the cell and this initiates apoptosis, as indicated by phosphatidylserine (PS) exposure on the external cell membrane (pink star shapes); membrane integrity becomes disrupted which permits experimentally added propidium iodide (PI) to enter the cell (top right) and indicates that the cell has entered the later stages of apoptosis. Schistosomes (adult male and female depicted, left) expressing SmNPP5 on their external surfaces (or recombinant SmNPP5 by itself) can cleave extracellular NAD (to generate NMN and AMP). This removes the substrate for ART2, preventing P2X7R ribosylation and blocking Treg apoptosis. Such an impact *in vivo* could promote Treg survival and help create a more immunologically hospitable environment for intravascular schistosomes. This would ultimately promote the survival of these debilitating global pathogens.
